# Sudanese medical students’ satisfaction with online learning and its association with their psychological distress: a cross-sectional study

**DOI:** 10.1186/s12909-025-07228-1

**Published:** 2025-05-14

**Authors:** Muhannad Bushra Masaad Ahmed, Ahmed Balla M. Ahmed, Moram Elfadel Abdelrhaman Gasmalha, Olla Zuhair Elamin Abdalla, Safinat Hassan Mohammed Ahmed, Blsam Abdelmoneam Ahmed Mohammed, Rabaa Alamein Omer Ebrahim, Maab Hisham Saeed Abdeen, Esraa Obeid Mohammed Babiker, Tasneem Abdelgader Abdelrhman Ahmed, Alkhansa Ahmed Osman Ahmed, Doaa Salaheldin Mohamed Ahmed, Muhammad Mubashir, Sohaib Mohammed Mokhtar Ahmed

**Affiliations:** 1 Faculty of Medicine and Health Sciences, University of Gadarif, Gadarif, Sudan; 2 Faculty of Medicine, University of Khartoum, Al-Qasr Street, PO Box: 102, Khartoum, 11111 Sudan; 3 Faculty of Medicine and Health Sciences, Omdurman Islamic University, Omdurman, Sudan; 4 Faculty of Medicine and Health Sciences, Red Sea University, Port Sudan, Sudan; 5 Faculty of Medicine, Elrazi University, Khartoum, Sudan; 6 Liaquat National Hospital and Medical College, Karachi, Pakistan

**Keywords:** Students' satisfaction, Depression, Anxiety, Stress, Online learning, War, Sudan

## Abstract

**Background:**

Online learning has emerged as an alternative to continuing higher education during the ongoing conflict in Sudan. Despite its numerous benefits, online learning is often associated with challenges like stress, anxiety, and depression. Satisfaction plays a pivotal role in shaping students’ perceptions of online education quality and their mental well-being, especially under adverse conditions like conflict. Hence, this study aimed to evaluate the satisfaction of Sudanese medical students with online learning and its association with psychological distress issues, including anxiety, depression, and stress, during the current Sudan war.

**Methods:**

A cross-sectional study was conducted among Sudanese medical students who transitioned to online learning during the conflict. Satisfaction with online learning was assessed using a validated questionnaire consisting of eight dimensions, while depression symptoms were measured with the Patient Health Questionnaire-9, anxiety with the Generalized Anxiety Disorder-7, and perceived stress with the Perceived Stress Scale-10. Associations between demographic factors and key outcomes were analyzed using independent t-tests, chi-square tests, and one-way ANOVA, with statistical significance set at *p* < 0.05.

**Results:**

Among the 1,891 medical students surveyed, the mean satisfaction score for online learning was 26.2 out of 40. Mild to moderate depression was reported by 59.4% of students, while 62% experienced mild to moderate anxiety, and 21% reported high stress levels. Lower satisfaction was significantly associated with higher levels of depression, anxiety, and stress (all *p* < 0.001).

**Conclusion:**

Sudanese medical students reported positive satisfaction with online learning during the conflict, yet this was accompanied by high levels of depression, anxiety, and stress. These findings highlight the need for integrated mental health support and improved digital access to enhance online education. Addressing these challenges is essential to ensuring student well-being and sustaining quality education in conflict settings. Further research across different settings can help develop more effective interventions.

## Background

Online learning has emerged as a potential solution, maintaining educational continuity during conflict situations [1]. Online learning is defined as instruction delivered electronically through multimedia and internet platforms or applications. It is often used interchangeably with terms like web-based learning, e-learning, and internet-based learning [2]. A key determinant of the effectiveness of online learning is student satisfaction, which reflects the quality of the educational program and its ability to meet students’ expectations [3]. However, levels of satisfaction vary widely. A previous study conducted in Khartoum State during the COVID-19 pandemic revealed that only 11.6% of medical students were satisfied with their online education experience, 52.4% were dissatisfied, and 36% were neutral [4].

Despite its advantages, online learning presents considerable challenges. Technical issues, limited internet access, and insufficient digital skills among both students and instructors hinder its effectiveness [5]. In conflict settings, these problems are worsened by unreliable network connections, dependence on mobile devices, frequent power outages, and internet disruptions, making it difficult for students to engage in lessons and leading to frustration and academic struggles [6]. These technical and environmental barriers not only disrupt education but also take a toll on students’ mental well-being, contributing to heightened levels of psychological distress [6–10]. Psychological distress is a broad term encompassing a spectrum of mental health conditions, from mild emotional strain to clinically diagnosed disorders such as depression, anxiety, stress, and posttraumatic stress disorder (PTSD) [11]. The impact of online learning challenges on students’ mental health has been well-documented. For instance, a study conducted during the Ukrainian war found that online learning triggered feelings of uncertainty, hesitation, and a lack of psychological safety among students. Additionally, 13% of participants reported anxiety and worry due to technical issues, while 22% faced depression, reduced interest, lack of focus, and limited personal connections [12]. These mental health challenges are not only disruptive but can also have significant consequences on students’ academic performance. A study conducted among Afghan female students, for example, found a significant correlation between academic achievement, anxiety, and sleep disturbances [13].

Previous research on the relationship between online learning satisfaction and psychological distress has shown mixed results. A study among nursing students in Saudi Arabia found no significant association between the two variables [14], while research conducted in Romania among medical undergraduates reported that higher levels of psychopathology were linked to lower satisfaction with online learning [15]. However, these studies were conducted in relatively stable environments.

Despite growing research on online learning and student well-being, there is a lack of evidence on how ongoing conflict affects the satisfaction and mental health of medical students using online education in Sudan. War-related stressors have already contributed to high levels of anxiety, depression, and post-traumatic stress disorder among these students [16], and the added strain of online learning in such conditions may further worsen their psychological state. Yet, no study has examined the link between student satisfaction with online learning and psychological distress in this unique context. This gap limits the ability to develop effective educational strategies and targeted mental health interventions during crises.

This study had the following objectives:

1. Assess Sudanese medical students’ satisfaction with online learning during the ongoing Sudan war.
2. Evaluate the prevalence of psychological distress (anxiety, depression, and stress) among these students.
3. Examine the association between satisfaction with online learning and psychological distress.

The following null hypotheses were formulated to guide the study:

### Hypothesis 1

There is no significant association between satisfaction with online learning and anxiety levels among Sudanese medical students during the ongoing Sudan war.

### Hypothesis 2

Satisfaction with online learning does not have a significant relationship with depression among Sudanese medical students during the ongoing Sudan war.

### Hypothesis 3

Satisfaction with online learning is not significantly correlated with stress among Sudanese medical students during the ongoing Sudan war.

## Methods

### Study design and setting

This cross-sectional study was conducted among medical students from all medical schools in Sudan in November 2024 to assess the satisfaction of Sudanese medical students with their online learning experience and its relationship with psychological distress, including anxiety, depression, and stress, during the war. This study focuses solely on the theoretical component of the medical curriculum, which includes lectures, discussions, and assessments conducted online during the conflict. In contrast, clinical training, which requires hands-on patient care and practical experience, continues in person as it cannot be effectively delivered remotely. Since clinical training was not conducted online, it was not examined in this study.

### Study population

Participants in this study were undergraduate medical students, aged 18 and above, currently enrolled in public or private medical schools in Sudan, and experienced online study during the study period. Eligibility required students to have access to a smartphone or laptop with internet connectivity and to provide voluntary consent to participate. Those who did not provide informed consent were excluded from the study.

### Sampling

We estimated the sample size using the Cochrane formula, given the unavailability of official population records of medical students in Sudan. Assuming a population proportion of 50%, a margin of error of 5%, and a confidence interval of 95%, the minimum sample size required was calculated to be 385 participants. To enhance the study’s validity and generalizability, it was intended to approach as many students as possible to gather maximum data during the data collection period. The final sample size reached was 1,891 medical students, ensuring broader representation and robustness in the findings.

Convenience sampling was employed in this study due to the challenges posed by the ongoing conflict in Sudan, which made accessing official student records impossible. The war has disrupted institutional operations, including data management systems, resulting in the unavailability of updated, centralized records of medical students. Additionally, the conflict has led to a fragmented educational system, with students dispersed across different regions and institutions, further complicating data access.

### Data collection tools

An online questionnaire using Google Forms was distributed among medical students via popular social media platforms such as WhatsApp, Telegram, and Facebook. These platforms were selected because they are widely utilized in Sudan. This method offers several advantages, such as the ability to gather large amounts of data efficiently, cost-effectiveness, and timely data collection [17]. Nonetheless, it may introduce certain biases and limit the sample’s representativeness, as students without stable internet access or those less engaged on social media could be underrepresented.

The questionnaire comprised three sections. The first section collected participants’ sociodemographic information. The second section assessed satisfaction with e-learning using a validated scale [18]. The third section evaluated psychological distress, including depression, anxiety, and stress, all measured using standardized scales.

### Variables and measures

This study assessed several variables categorized as independent and outcome variables to evaluate the relationship between e-learning satisfaction and psychological distress among Sudanese medical students.

### A. Independent variables

#### Sociodemographic factors

Data were collected through eight questions covering:

Age: Reported in years.

Gender: Male or female.

Region of residence: Categorized as Arabic Gulf (Saudi Arabia, UAE, Oman, Kuwait, etc.), Central Africa (Uganda, Tanzania, etc.), North Africa (Egypt, Libya, etc.), Sudan, and other. Notably, students residing outside Sudan were still registered in Sudanese medical colleges. Many had relocated due to the war but continued their education online, with universities providing centers for training, some of the theoretical courses, and exams in other countries.

Year of study: From the first to the sixth year.

Living situation: Living alone, with family, with relatives, or with roommates.

Employment status: Defined as employed (including full-time or part-time), freelancer (engaged in gig or contract work), or unemployed (not engaged in any paid work).

Device access for online learning: Categorized as none, one, two, or three or more devices.

Family socioeconomic status: Self-reported and categorized as high, upper-middle, middle, lower-middle, or low.

### B. Outcome variables

#### E-learning satisfaction

E-learning satisfaction was measured using a validated scale consisting of eight dimensions: satisfaction with assessment, course content and organisation, instructor, learning environment and teaching methods, learning resources, quality of delivery, student contribution, and tutorials. Responses were recorded on a 5-point Likert scale ranging from 1 (Very dissatisfied) to 5 (Very satisfied). Mean scores were used to assess satisfaction for each dimension, and the instrument demonstrated excellent reliability with a Cronbach’s alpha of 0.986 [18].

####  Psychological distress

Depression: Assessed using the PHQ-9 (Patient Health Questionnaire-9), a screening tool for depressive symptoms over the past two weeks. This nine-item scale measures depressive symptoms, with each item scored from 0 (Not at all) to 3 (Nearly every day), resulting in total scores ranging from 0 to 27. Cut-points of 5, 10, 15, and 20 represent mild, moderate, moderately severe, and severe depression, respectively [19]. Internal consistency of the PHQ-9 has been shown to be high, with Cronbach’s alpha of 0.89 [20]. Anxiety: Measured using the GAD-7 (Generalized Anxiety Disorder-7), a screening tool for anxiety symptoms over the past two weeks. This seven-item scale evaluates anxiety symptoms, with each item scored from 0 (Not at all) to 3 (Nearly every day), and total scores ranging from 0 to 21. Scores of 5, 10, and 15 represent mild, moderate, and severe anxiety, respectively. The GAD-7 shows good test-retest reliability and excellent internal consistency [21, 22]. Perceived stress: Measured using the PSS-10 (Perceived Stress Scale-10), a ten-item scale assessing stress levels over the past month. Items focus on feelings of control, overwhelm, and nervousness, scored from 0 (Never) to 4 (Very often). Total scores range from 0 to 40, categorized as low stress (0–13), moderate stress (14-26), and high stress (27-40). The PSS-10 has been shown to have good internal consistency, adequate test-retest reliability, and good concurrent validity. The Perceived Stress Scale is not a diagnostic instrument. It evaluates the degree to which an individual has perceived life as unpredictable, uncontrollable, and overloading over the previous month [23, 24].

The questionnaire was prepared in English, which poses no challenges, as English is the primary language of instruction in Sudanese medical colleges [25]. The survey was conducted anonymously, and no incentives were provided for participation.

### Data management

Statistical analysis was conducted using Statistical Package for the Social Sciences (SPSS) software version 27. Descriptive statistics, including means, standard deviations, medians, and percentages, were used to summarize sociodemographic characteristics, satisfaction scores, and psychological distress outcomes (depression, anxiety, and stress). Inferential statistical methods, such as independent samples t-tests, chi-square tests, and one-way ANOVA, were employed to examine associations between demographic factors and key study outcomes, including satisfaction levels and psychological distress measures.

Correlation analyses were conducted to explore relationships between satisfaction with online learning and psychological distress outcomes. Multiple linear regression models were conducted, treating satisfaction as the dependent variable when analyzed with mental health and demographic factors. In models examining mental health outcomes, including depression, anxiety, and stress, each was considered the dependent variable with demographic factors as independent variables. Estimates, standard errors (SE), t-values, and p-values were reported to indicate the strength and significance of associations. All statistical tests were two-tailed, with statistical significance set at *p* < 0.05.

## Results

### Socio-demographic features

The study sample included 1,891 medical students with a mean age of 22.5 years (SD = 2.4), ranging from 18 to 45 years. Female participants comprised 63.2% of the sample, with males representing 36.8%. Most participants currently residing in Sudan (61.6%), followed by the Arabic Gulf countries (19.7%), North Africa (15.9%), Central Africa (1.7%), and other regions (1.1%). Representation across academic years was varied, with the highest participation from fifth-year students (24.4%) and the lowest from first- and sixth-year students (9.3% each). The majority (75.7%) lived with family, while others resided with roommates (12.3%), relatives (6.3%), or alone (5.6%). Employment data showed that 80.0% were unemployed, 11.5% were employed, and 8.6% were freelancers. Device access for online learning varied, with 47.2% having one device, 39.3% having two, 8.0% having three or more, and 5.5% reporting no device. Economic status was mainly middle class (63.4%), with smaller proportions in the upper-middle (12.9%), lower-middle (14.9%), low (6.6%), and high (2.2%) economic status brackets (Table 1).

**Table 1:** Socio-demographic and economic characteristics of study participants

	Overall (*N* = 1891)
**Age**	
Mean (SD)	22.5 (2.4)
Range	18.0–45.0
**Gender**	
Female	1195 (63.2%)
Male	696 (36.8%)
**Region of Residence**	
Arabic Gulf (Saudi Arabia, UAE, Oman, Kuwait, etc.)	373 (19.7%)
Central Africa (Uganda, Tanzania, etc.)	33 (1.7%)
North Africa (Egypt, Libya, etc.)	301 (15.9%)
Sudan	1164 (61.6%)
Other	20 (1.1%)
**Year of Study**	
First year	176 (9.3%)
Second year	308 (16.3%)
Third year	398 (21.0%)
Fourth year	372 (19.7%)
Fifth year	462 (24.4%)
Sixth year	175 (9.3%)
**Living Situation**	
Living alone	106 (5.6%)
Living with family	1432 (75.7%)
Living with relatives	120 (6.3%)
Living with roommates	233 (12.3%)
**Employment Status**	
Employed	217 (11.5%)
Freelancer	162 (8.6%)
Unemployed	1512 (80.0%)
**How many devices do you have access to for online learning?**	
None	104 (5.5%)
One	892 (47.2%)
Two	744 (39.3%)
Three or more	151 (8.0%)
**Economic Status**	
High	42 (2.2%)
Upper-middle	244 (12.9%)
Middle	1198 (63.4%)
Lower-middle	282 (14.9%)
Low	125 (6.6%)

### Satisfaction with online learning

**Fig. 1:**
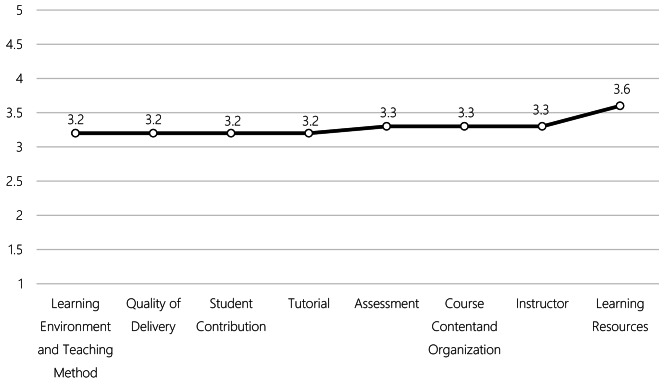
Average satisfaction ratings of various aspects of online learning among medical students

The mean satisfaction score for online learning was 26.2 (SD = 6.5) out of 40, with positive satisfaction across domains such as course content, instructor performance, and resources (Fig. 1). An independent samples t-test revealed that female students had a slightly lower mean satisfaction score (25.8, SD = 6.53) compared to male students (26.8, SD = 6.28), with a statistically significant difference (*p* = 0.001). A one-way ANOVA also indicated significant differences in satisfaction across study years (χ² = 26.8, *p* < 0.001), where fifth-year students reported lower satisfaction than sixth-year students (*p* = 0.001). Living arrangements significantly influenced satisfaction levels (χ² = 30.8, *p* < 0.001), with students living alone reporting lower satisfaction than those living with family, relatives, or roommates. Employment status was similarly impactful; employed students reported higher satisfaction than both freelancers and unemplostudents (χ² = 31.5, *p* < 0.001). Lastly, socio-economic status (SES) significantly influenced satisfaction (χ² = 18.8, *p* < 0.001), with higher SES groups reporting greater satisfaction. The linear regression analysis reveal that several factors significantly influenced satisfaction with online learning among medical students. Age was positively associated with satisfaction (*p* = 0.002), with older students reported higher levels of satisfaction. Living arrangements also impacted satisfaction where students living alone reported lower satisfaction compared to those living with family, relatives, or roommates (all *p* < 0.001). Employment status further influenced satisfaction, as employed students had higher satisfaction than freelancers (*p* = 0.015) and unemployed students (*p* < 0.001).

### Depression

Depression levels, assessed via the PHQ-9 scale, revealed a median depression score of 10 (IQR = 8.0, range: 0–27). Among the participants, 12.5% had minimal symptoms (scores of 0–4), 30.9% experienced mild symptoms (scores of 5–9), 28.5% moderate, 19.4% moderately severe, and 8.7% severe depression (scores of 20–27). (Table 2). Age, gender, employment status, and economic status showed significant associations with depressive symptoms. Students with depressive symptoms had a slightly younger mean age than those without (F = 12.07, *p* < 0.013), with males reporting fewer symptoms than females (Χ² = 11.13, *p* < 0.012). Unemployed individuals are more likely to have depressive symptoms, while employed and freelancers are less likely (X² = 9.08, *p* = 0.012). Economic status was also significant, with students in lower-middle SES more likely to report symptoms than those in higher categories (Χ² = 21.11, *p* < 0.012) (Table 3). The regression model results show that gender was a significant predictor of PHQ-9 score, with males having a lower score than females (Estimate = − 0.822, SE = 0.2964, *p* = 0.006). Other predictors, including age, region of residence, year of study, living situation, employment status, and the number of devices for online learning, were not significant.

**Table 2:** Distribution of depression, anxiety, and stress levels among participants

		Frequency	Percentage %
**Depression**	Minimal (0–4)	237	12.5%
	Mild (5–9)	585	30.9%
	Moderate (10–14)	538	28.5%
	Moderately Severe (15–19)	366	19.4%
	Severe (20–27)	165	8.7%
**Anxiety**	Normal (0–4)	475	25.1%
	Mild (5–9)	710	37.5%
	Moderate (10–14)	460	24.3%
	Severe (15–21)	246	13.0%
**Stress Symptoms**	Low Perceived Stress (0–13)	697	36.9%
	Moderate Perceived Stress (14–26)	799	42.3%
	High Perceived Stress (27–40)	395	20.9%

**Table 3:** Comparison of socio-demographic and economic factors by depression status

	*N*	No Depressive Symptoms (PHQ−9 < 15)	Depressive Symptoms (PHQ−9 > = 15)	Test Statistic
		(*N* = 1360)	(*N* = 531)	
**Age**	1891	21.0 **23.0** 24.0	21.0 **22.0** 24.0	F_1,1889_=12.07, *P* < 0.01^1^
**Gender: Male**	1891	532 (39.12%)	164 (30.89%)	Χ^2^ =11.13, *P* < 0.01^2^
**Region of Residence**	1891			Χ^2^ =1.81, *P* = 0.77^2^
Arabic Gulf (Saudi Arabia, UAE, Oman, Kuwait, etc.)		271 (19.91%)	102 (19.21%)	
Central Africa (Uganda, Tanzania, etc.)		21 (1.54%)	12 (2.26%)	
North Africa (Egypt, Libya, etc.)		219 (16.13%)	82 (15.44%)	
Sudan		836 (61.53%)	328 (61.76%)	
Other		13 (0.96%)	7 (1.32%)	
**Year of Study**	1891			Χ^2^ =10.03, *P* = 0.07^2^
Fifth year		341 (25.00%)	121 (22.78%)	
First year		124 (9.12%)	52 (9.79%)	
Fourth year		262 (19.26%)	110 (20.71%)	
Second year		208 (15.29%)	100 (18.84%)	
Sixth year		140 (10.29%)	35 (6.59%)	
Third year		285 (20.98%)	113 (21.28%)	
**Living Situation**	1891			Χ^2^ =6.08, *P* = 0.11^2^
Living alone		86 (6.32%)	20 (3.77%)	
Living with family		1022 (75.00%)	410 (77.23%)	
Living with relatives		81 (5.94%)	39 (7.34%)	
Living with roommates		171 (12.57%)	62 (11.67%)	
**Employment Status**	1891			Χ^2^ =9.08, *P* = 0.01^2^
Employed		168 (12.35%)	49 (9.22%)	
Freelancer		128 (9.41%)	34 (6.40%)	
Unemployed		1064 (78.24%)	448 (84.36%)	
**Economic Status**	1891			Χ^2^ =21.11, *P* < 0.01^2^
High		35 (2.57%)	7 (1.32%)	
Low		81 (5.94%)	44 (8.28%)	
Lower-middle		177 (13.03%)	105 (19.75%)	
Middle		882 (64.91%)	316 (59.47%)	
Upper-middle		185 (13.61%)	59 (11.11%)	

^1^Wilcoxon. ^2^Pearson.

### Anxiety

Anxiety levels were measured using the GAD-7 scale, with a median score of 8 (IQR = 8.0, range: 0–21). Scores were classified into normal (25.1%), mild (37.5%), moderate (24.3%), and severe (13.0%) categories (Table 2). Age, gender, living situation, and economic status were significantly associated with anxiety symptoms. Students with anxiety were slightly older than those without (F = 5.02, *p* = 0.033), and females were more likely than males to report symptoms (Χ² = 16.37, *p* < 0.012). Anxiety was more prevalent among students living with family (Χ² = 8.70, *p* = 0.032) and among those from lower-middle and upper-middle economic status brackets (Χ² = 15.94, *p* < 0.012) (Table 4). The regression model results for the GAD-7 score revealed significant associations with age, gender, and year of study. Age was negatively associated with the GAD-7 score (Estimate = − 0.150, SE = 0.0698, *p* = 0.031). Gender was also a significant predictor, with males having lower scores than females (Estimate = − 1.240, SE = 0.2716, *p* < 0.001). Additionally, students in their sixth year had significantly lower GAD-7 scores compared to fifth-year students (Estimate = − 1.370, SE = 0.4844, *p* = 0.005). Other predictors, including region of residence, living situation, employment status, and access to online learning devices, were not significantly associated with GAD-7 scores.

**Table 4:** Comparison of socio-demographic and economic factors by anxiety status

	*N*	No Anxiety Symptoms (GAD−7 < 15)	Anxiety Symptoms (GAD−7 ≥ 15)	Test Statistic
		(*N* = 1645)	(*N* = 246)	
**Age**	1891	21.0 **22.0** 24.0	21.0 **22.0** 23.0	F_1,1889_=5.02, *P* = 0.03^1^
**Gender: Male**	1891	634 (38.5%)	62 (25.2%)	Χ^2^ =16.37, *P* < 0.01^2^
**Region of Residence**	1891			Χ^2^ =1.60, *P* = 0.81^2^
Arabic Gulf (Saudi Arabia, UAE, Oman, Kuwait, etc.)		325 (19.7%)	48 (19.5%)	
Central Africa (Uganda, Tanzania, etc.)		30 (1.8%)	3 (1.2%)	
North Africa (Egypt, Libya, etc.)		259 (15.7%)	42 (17.1%)	
Sudan		1015 (61.7%)	149 (60.6%)	
Other		16 (1.0%)	4 (1.6%)	
**Year of Study**	1891			Χ^2^ =6.92, *P* = 0.23^2^
Fifth year		395 (24.0%)	67 (27.2%)	
First year		157 (9.5%)	19 (7.7%)	
Fourth year		314 (19.1%)	58 (23.6%)	
Second year		268 (16.3%)	40 (16.3%)	
Sixth year		159 (9.7%)	16 (6.5%)	
Third year		352 (21.4%)	46 (18.7%)	
**Living Situation**	1891			Χ^2^ =8.70, *P* = 0.03^2^
Living alone		96 (5.8%)	10 (4.1%)	
Living with family		1229 (74.7%)	203 (82.5%)	
Living with relatives		105 (6.4%)	15 (6.1%)	
Living with roommates		215 (13.1%)	18 (7.3%)	
**Employment Status**	1891			Χ^2^ =4.87, *P* = 0.09^2^
Employed		198 (12.0%)	19 (7.7%)	
Freelancer		144 (8.8%)	18 (7.3%)	
Unemployed		1303 (79.2%)	209 (85.4%)	
**Economic Status**	1891			Χ^2^ =15.94, *P* < 0.01^2^
High		37 (2.2%)	5 (2.0%)	
Low		108 (6.6%)	17 (6.9%)	
Lower-middle		228 (13.9%)	54 (22.0%)	
Middle		1067 (64.9%)	131 (53.3%)	
Upper-middle		205 (12.5%)	39 (15.9%)	

^1^Wilcoxon. ^2^Pearson.

### Perceived stress

Perceived stress levels (PSS) with a median score of 18 (IQR = 15.0, range: 0–40) indicated that 36.9% of students experienced low stress (scores of 0–13), 42.3% moderate (scores of 14–26), and 20.9% high (scores of 27–40) (Table 2). Significant associations were found between perceived stress and age, gender, residence, living situation, employment, and economic status. Older students tended to report higher stress (F = 5.71, *p* < 0.011), with females reporting higher stress than males (Χ² = 45.38, *p* < 0.012). Students residing in North Africa were more likely to experience high stress (Χ² = 19.20, *p* = 0.012). Living with family correlated with higher stress levels (Χ² = 18.50, *p* = 0.012), while students in lower-middle SES showed a higher prevalence of stress (Χ² = 34.07, *p* < 0.012) (Table 5). The regression model results for the PSS score revealed significant associations with age, gender, employment status, and the number of devices for online learning. Age was negatively associated with the PSS score (Estimate = − 0.379, SE = 0.128, *p* = 0.003). Males had significantly lower scores compared to females (Estimate = − 2.907, SE = 0.499, *p* < 0.001). Being unemployed was associated with higher PSS scores compared to being employed (Estimate = 2.885, SE = 0.740, *p* < 0.001). Other predictors, including region of residence, year of study, and living situation, were not significantly associated with PSS scores.

**Table 5:** Comparison of socio-demographic and economic factors by perceived stress level

	*N*	Low Perceived Stress	High Perceived Stress	Moderate Perceived Stress	Test Statistic
		(*N* = 697)	(*N* = 395)	(*N* = 799)	
**Age**	1891	21.0 **23.0** 24.0	21.0 **22.0** 23.0	21.0 **22.0** 24.0	F_2,1888_=5.71, *P* < 0.01^1^
**Gender: Male**	1891	317 (45.5%)	101 (25.6%)	278 (34.8%)	Χ^2^ =45.38, *P* < 0.01^2^
**Region of Residence**	1891				Χ^2^ =19.20, *P* = 0.01^2^
Arabic Gulf (Saudi Arabia, UAE, Oman, Kuwait, etc.)		133 (19.1%)	71 (18.0%)	169 (21.1%)	
Central Africa (Uganda, Tanzania, etc.)		14 (2.0%)	11 (2.8%)	8 (1.0%)	
North Africa (Egypt, Libya, etc.)		90 (12.9%)	69 (17.5%)	142 (17.8%)	
Sudan		448 (64.3%)	241 (61.0%)	475 (59.5%)	
Other		12 (1.7%)	3 (0.8%)	5 (0.6%)	
**Year of Study**	1891				Χ^2^ =11.71, *P* = 0.30^2^
Fifth year		168 (24.1%)	86 (21.8%)	208 (26.0%)	
First year		67 (9.6%)	36 (9.1%)	73 (9.1%)	
Fourth year		124 (17.8%)	82 (20.8%)	166 (20.8%)	
Second year		110 (15.8%)	66 (16.7%)	132 (16.5%)	
Sixth year		80 (11.5%)	34 (8.6%)	61 (7.6%)	
Third year		148 (21.3%)	91 (23.0%)	159 (19.9%)	
**Living Situation**	1891				Χ^2^ =18.50, *P* = 0.01^2^
Living alone		58 (8.3%)	13 (3.3%)	35 (4.4%)	
Living with family		503 (72.1%)	314 (79.5%)	615 (77.0%)	
Living with relatives		45 (6.5%)	26 (6.6%)	49 (6.1%)	
Living with roommates		91 (13.1%)	42 (10.6%)	100 (12.5%)	
**Employment Status**	1891				Χ^2^ =41.90, *P* < 0.01^2^
Employed		119 (17.1%)	33 (8.4%)	65 (8.1%)	
Freelancer		72 (10.3%)	32 (8.1%)	58 (7.3%)	
Unemployed		506 (72.5%)	330 (83.6%)	676 (84.5%)	
**Economic Status**	1891				Χ^2^ =34.07, *P* < 0.01^2^
High		21 (3.0%)	7 (1.8%)	14 (1.8%)	
Low		50 (7.2%)	27 (6.8%)	48 (6.0%)	
Lower-middle		79 (11.3%)	91 (23.0%)	112 (14.0%)	
Middle		460 (66.1%)	229 (58.1%)	509 (63.7%)	
Upper-middle		87 (12.5%)	41 (10.4%)	116 (14.5%)	

^1^Kruskal-Wallis. ^2^Pearson.

### Association between satisfaction and mental health outcomes

Reduced satisfaction is weakly associated with higher depressive symptoms (PHQ-9; rho = -0.286, *p* < 0.001) and weakly linked to increased perceived stress (PSS; rho = -0.382, *p* < 0.001). Additionally, lower satisfaction shows a weaker but significant association with elevated anxiety levels (GAD-7; rho = -0.213, *p* < 0.001). Furthermore, the regression analysis demonstrates a significant predictive relationship between satisfaction with online learning and mental health outcomes. In the GAD-7 model, satisfaction scores are significantly negatively associated with anxiety levels (Estimate = -0.206, SE = 0.0187, t = -11.0, *p* < 0.001), indicating that lower satisfaction is linked to higher anxiety. For depression, as measured by the PHQ-9, the satisfaction score similarly exhibits a significant negative relationship (Estimate = -0.287, SE = 0.0199, t = -14.4, *p* < 0.001), suggesting that dissatisfaction contributes to greater depressive symptoms. The strongest association is observed in the PSS model, where satisfaction scores significantly predict perceived stress (Estimate = -0.598, SE = 0.0331, t = -18.1, *p* < 0.001), with lower satisfaction being associated with markedly higher stress levels.

## Discussion

During the Sudan conflict, medical colleges in affected areas adopted online learning as an alternative teaching method. While e-learning provides a safe environment, engaging platforms, and potential for quality education [26], its implementation in a low-income country like Sudan has faced significant challenges and psychological impacts on students. Student satisfaction with online learning has been a key concern, as varying levels of satisfaction may influence engagement and overall learning experiences. This study aimed to assess Sudanese medical students’ satisfaction with online learning and its relationship with psychological distress, encompassing anxiety, depression, and stress, during the ongoing Sudan war.

Satisfaction levels with online learning were generally positive. However, lower satisfaction was associated with higher levels of depression, anxiety, and perceived stress, highlighting the impact of online education on students’ mental well-being.

Satisfaction levels with online learning in this study were above average, slightly lower than a comparable study in Pakistan [18]. Positive satisfaction was observed across domains, with the highest scores in the learning resources domain. Female students reported slightly lower satisfaction with online learning compared to male students, differing from findings in a previous study [27]. This may be due to gender-specific challenges in Sudan during the war, including greater household and caregiving responsibilities, as well as mobility restrictions that limit access to study spaces and starlink, the main internet source in many areas during the conflict. Higher socioeconomic status (SES) groups reported greater satisfaction with online learning, likely due to better access to stable internet, suitable study environments, and digital devices, which facilitate a smoother learning experience. Linear regression revealed that satisfaction with online learning was influenced by age, living arrangements, and employment status, with older students, those living with family or roommates, and employed students reporting higher satisfaction. Older students may have greater maturity, self-discipline, and experience with independent learning, which aligns with a previous study from Nigeria [28]. Similarly, students living with their families likely benefit from the positive effects of family support on learning engagement, as supported by a previous study [29]. Additionally, employed students may experience greater financial stability, allowing them to afford better devices and reliable internet access, which can contribute to higher satisfaction with online learning.

Our findings revealed that slightly more than half of participants experienced mild to moderate depression symptoms, significantly higher than those reported in a study during the Ukrainian war [12]. This difference reflects the unique challenges faced by Sudanese students, including limited internet access, frequent power outages, and the strain of pursuing education amid a humanitarian crisis. Anxiety symptoms were also higher among participants compared to Ukrainian students [12], revealing the compounded effects of Sudan’s status as a low-income country. The severity of the conflict further contributes to these differences. The war in Sudan has led to widespread displacement, destruction of infrastructure and health system, and prolonged uncertainty, severely disrupting daily life [30]. In contrast, despite ongoing conflict, Ukraine has maintained more structured support systems and international assistance [31], which may have mitigated some of the psychological toll on students. Cultural factors also play a role in shaping mental health outcomes. In Sudan, mental health stigma remains a significant barrier, preventing many individuals from seeking professional help [32]. Traditional coping mechanisms, such as reliance on family and community networks, may provide some support but are often insufficient in addressing severe psychological distress. Meanwhile, Ukraine has a more established framework for mental health awareness and care, which may contribute to lower reported rates of anxiety and depression among students [33]. A key contributing factor to these disparities is the lack of efforts to mitigate the psychological effects of war in Sudan. Unlike Ukraine, where the government established the Inter-agency Coordination Council on Mental Health Protection and Psychological Assistance to support those affected by the conflict [34], Sudan has seen no such initiatives. Even before the war, the country faced a severe shortage of mental health resources, with only 0.08 psychiatrists per 100,000 people in 2020, further exacerbating the current crisis [35]. Both depression and anxiety levels were lower than the baseline reported among Khartoum State residents at the onset of the war [36]. This may be due to the fact that a third of the medical students currently reside in safer locations outside Sudan, meaning they were not directly affected by the war, unlike the population in the Khartoum study.

Perceived stress levels indicated that nearly two-thirds of participants experienced moderate to high stress, slightly lower than rates observed in Bangladeshi students during COVID−19 [37] and those observed among Sudanese pharmacy students in physical learning settings amid the pandemic [38]. This lower stress levels compared to Sudanese pharmacy students may arise from differences between stressors in online and physical learning, as online learning reduces some logistical burdens. These three psychological distress outcomes—depression, anxiety, and stress—were lower than those observed in Palestinian young adults during the Gaza war [39], likely due to the greater severity of the conflict in Gaza, which caused massive destruction to the healthcare system and infrastructure.

Lower satisfaction levels in our study were associated with higher levels of depression, anxiety, and perceived stress, aligning with findings from research in Malaysia, Romania, and a study conducted in Portugal during the COVID-19 pandemic [15, 40, 41]. Increasing satisfaction with e-learning through improved course design, enhanced interaction, and accessible resources can foster a more positive academic experience. This, in turn, can alleviate mental health symptoms, as greater satisfaction reduces stress and promotes engagement, ultimately improving overall psychological well-being. Integrating mental health support within online platforms can further reinforce this cycle of satisfaction and mental health improvement.

Psychological distress was significantly influenced by gender, age, economic status, and employment status. Females exhibited higher levels of depression, anxiety, and stress compared to males, as confirmed by the regression models, which identified gender as a significant predictor for all three distress measures. This aligns with a previous study showing that female students in Iraq and Lebanon faced greater challenges with online learning than their male counterparts [42].

Depression was more prevalent among younger students from low-income backgrounds. A U.S. study found that young women from low-income households consistently experience higher depression rates, highlighting that these issues are multifaceted and extend beyond educational formats [43]. Another study in the Middle East further supports this, showing that economic disparities hinder engagement with online learning materials [42].

In contrast, anxiety and stress levels were higher among older students, as indicated by the regression models. Older students, often balancing additional responsibilities such as family and work, exhibited greater psychological distress. This finding contrasts with studies from other regions, where older students tend to demonstrate greater confidence with technology [44].

Unemployment was a significant predictor of stress, with unemployed students experiencing notably higher stress levels. Additionally, limited access to online learning devices contributed to elevated stress, underscoring the impact of digital inequality on student well-being. Although the region of residence was not a significant factor in the regression models, students in North Africa reported higher stress levels, possibly due to displacement-related challenges. Displaced students frequently face instability and social isolation, making it difficult to adjust to new environments, secure housing, and maintain academic continuity under uncertain conditions. Furthermore, the loss of familiar support systems and concerns for family members still in conflict zones may further impact their mental health, contributing to the heightened stress levels observed among students in North Africa [45].

This study has several strengths. It is the first of its kind to assess medical students’ satisfaction with online learning during the Sudan conflict, providing critical insights into an unprecedented educational challenge. The methodology used offers the best approach to linking satisfaction with psychological distress, ensuring a comprehensive evaluation of students’ experiences. Additionally, the inclusion of Sudanese students who remained in the country and those who relocated abroad but continued their studies through online learning at Sudanese universities provides a broader understanding of satisfaction levels in different learning environments. Lastly, the findings have the potential for generalizability to similar war-affected settings, particularly those with shared cultural and educational contexts.

However, this study has some limitations. The reliance on convenience sampling, chosen due to the lack of official student records and logistical challenges, introduced potential biases by overrepresentation students who actively use social media platforms. This approach likely excluded students without internet access, reducing the representativeness of the findings and skewing the sample towards more accessible or urbanized groups. Socioeconomic status was based on self-reported family income, but participants’ perceptions of their socioeconomic status may vary, which could affect the findings. Additionally, social desirability bias, driven by stigma surrounding mental health in conflict zones, likely led to underreporting of psychological distress. This resulted in an underestimation of anxiety, depression, and stress. Emotional stress from the conflict likely influenced responses, making it challenging to isolate online learning experiences from the broader impact of the war. Additionally, the study focuses on immediate psychological impact and satisfaction, potentially overlooking the long-term academic and career effects of sustained online learning under such conditions. This study exclusively focuses on the theoretical component of medical education conducted online during the conflict. While clinical training continued physically in safer states, the study does not address its effect on students’ satisfaction and mental health. This limitation restricts the scope of the findings to the theoretical part of the curriculum and does not reflect the broader impact of the conflict on practical training.

## Conclusion

This study found that Sudanese medical students expressed positive satisfaction with online learning during the conflict. However, this satisfaction was significantly associated with high levels of psychological distress, including depression, anxiety, and stress. These findings highlight the urgent need to integrate mental health support into online education, particularly in crisis settings. Initiatives like Ukraine’s “Tell Me” platform demonstrate the value of accessible psychological support. Policies promoting social and financial support for students in conflict zones are also crucial. Additionally, improving online learning infrastructure, including better internet access and subsidized devices, can help sustain education in unstable environments. Future research should compare students in conflict and stable settings, incorporate qualitative insights, and expand across different conflict contexts to inform effective interventions.

## Data Availability

“The datasets used and/or analysed during the current study are available from the corresponding author on reasonable request.”

## Abbreviations

ANOVA: Analysis of Variance
PHQ-9: Patient Health Questionnaire-9
GAD-7: Generalized Anxiety Disorder-7
PSS-10: Perceived Stress Scale-10
SPSS: Statistical Package for the Social Sciences
SES: Socio-economic status
U.S: United States
COVID-19: Coronavirus disease 2019
